# Does Point of Care Ultrasound Improve Resuscitation Markers in Undifferentiated Hypotension? An International Randomized Controlled Trial From The Sonography in Hypotension and Cardiac Arrest in the Emergency Department (SHoC-ED) Series

**DOI:** 10.7759/cureus.9899

**Published:** 2020-08-20

**Authors:** Paul Atkinson, Luke Taylor, James Milne, Laura Diegelmann, Hein Lamprecht, Melanie Stander, David Lussier, Chau Pham, Ryan J Henneberry, Jacqueline Fraser, Michael Howlett, Jay Mekwan, Brian Ramrattan, Joanna Middleton, Daniel J Van Hoving, Mandy Peach, Tara Dahn, Sean Hurley, Kayla MacSween, Lucas Richardson, George Stoica, Sam Hunter, Jack P Atkinson, Paul Olszynski, Ankona Banerjee, David Lewis

**Affiliations:** 1 Emergency Medicine, Saint John Regional Hospital, Saint John, CAN; 2 Emergency Medicine, Dalhousie University, Saint John, CAN; 3 Family Medicine, Fraser Valley Health, Vancouver, CAN; 4 Emergency Medicine, University of Maryland, Baltimore, USA; 5 Emergency Medicine, Stellenbosch University, Cape Town, ZAF; 6 Emergency Medicine, Mediclinic, Cape Town, ZAF; 7 Emergency Medicine, University of Manitoba, Winnipeg, CAN; 8 Emergency Medicine, University of Manitoba, Winnipeg, CAN; 9 Emergency Medicine, Dalhousie University, Halifax, CAN; 10 Emergency Medicine, Horizon Health Network, Saint John, CAN; 11 Research Services, Horizon Health Network, Saint John, CAN; 12 Science, University of Ottawa, Ottawa, CAN; 13 Faculty of Science, Dalhousie University, Halifax, CAN; 14 Emergency Medicine, University of Saskatchewan, Saskatoon, CAN; 15 Medical Services, WorkSafeNB, Saint John, CAN

**Keywords:** emergency medicine, point of care ultrasound, shock, hypotension, critical care

## Abstract

Introduction

Point of Care Ultrasound (PoCUS) protocols are commonly used to guide resuscitation for patients with undifferentiated hypotension, yet there is a paucity of evidence for any outcome benefit. We undertook an international multicenter randomized controlled trial (RCT) to assess the impact of a PoCUS protocol on key clinical outcomes. Here we report on resuscitation markers.

Methods

Adult patients presenting to six emergency departments (ED) in Canada and South Africa with undifferentiated hypotension (systolic blood pressure (SBP) <100mmHg or a Shock Index >1.0) were randomized to receive a PoCUS protocol or standard care (control). Reported physiological markers include shock index (SI), and modified early warning score (MEWS), with biochemical markers including venous bicarbonate and lactate, at baseline and four hours.

Results

A total of 273 patients were enrolled, with data collected for 270. Baseline characteristics were similar for each group. Improvements in mean values for each marker during initial treatment were similar between groups: Shock Index; mean reduction in Control 0.39, 95% CI 0.34 to 0.44 vs. PoCUS 0.33, 0.29 to 0.38; MEWS, mean reduction in Control 2.56, 2.22 to 2.89 vs. PoCUS 2.91, 2.49 to 3.32; Bicarbonate, mean reduction in Control 2.71 mmol/L, 2.12 to 3.30 mmol/L vs. PoCUS 2.30 mmol/L, 1.75 to 2.84 mmol/L, and venous lactate, mean reduction in Control 1.39 mmol/L, 0.93 to 1.85 mmol/L vs. PoCUS 1.31 mmol/L, 0.88 to 1.74 mmol/L.

Conclusion

We found no meaningful difference in physiological and biochemical resuscitation markers with or without the use of a PoCUS protocol in the resuscitation of undifferentiated hypotensive ED patients. We are unable to exclude improvements in individual patients or in specific shock types.

## Introduction

We previously reported the outcomes for mortality and length of stay measures from an international randomized controlled trial for patients presenting to the emergency department (ED) with undifferentiated non-traumatic hypotension or shock who received treatment with or without point of care ultrasound (PoCUS) [1].

Although mortality rates were high, at around 25% in our study population, and in line with previously reported studies [2,3], we found no clear survival or length-of-stay benefits for patients in the PoCUS group. There was no difference in treatment received between groups.

Other markers of effective resuscitation, commonly used to assess disease severity, and to guide fluid administration and other interventions, include physiological measures such as the shock index (SI) and the modified early warning score (MEWS), as well as biochemical markers such as venous lactate and bicarbonate [4,5]. In this planned report of these secondary outcomes, we wished to assess if PoCUS use might lead to improved resuscitation over usual care, as evidenced by improved markers of resuscitation, which may indicate a physiological benefit. While PoCUS has been shown to assist in the assessment of fluid status, to improve early diagnosis, as well as in finding potential causes of hypotension [3,6,7], there are no prospective comparative studies looking at changes in key resuscitation markers in this patient population. 

We asked if the use of point of care ultrasound improved resuscitation markers in emergency department patients with undifferentiated hypotension, compared with standard care.

## Materials and methods

We completed an international, multi-centered, randomized controlled trial (RCT) of adult patients who presented to the emergency department with undifferentiated non-traumatic hypotension or shock (i.e. without a clearly evident etiology). Recruitment occurred in three centers in Canada and three in South Africa. Adult patients (aged 19 years or older) were screened after triage to identify either a sustained systolic blood pressure (SBP) <100 mmHg or a shock index >1.0. Patients were excluded due to the need for immediate PoCUS such as suspected ectopic pregnancy or aortic aneurysm, in addition to evidence of differentiated hypotension as indicated by cardiopulmonary resuscitation (CPR) or other advanced cardiac life support interventions; a history of significant recent trauma; acute myocardial infarction (AMI); another clear mechanism or etiology for the hypotension or shock such as gastrointestinal bleeding. 

**Figure 1:**
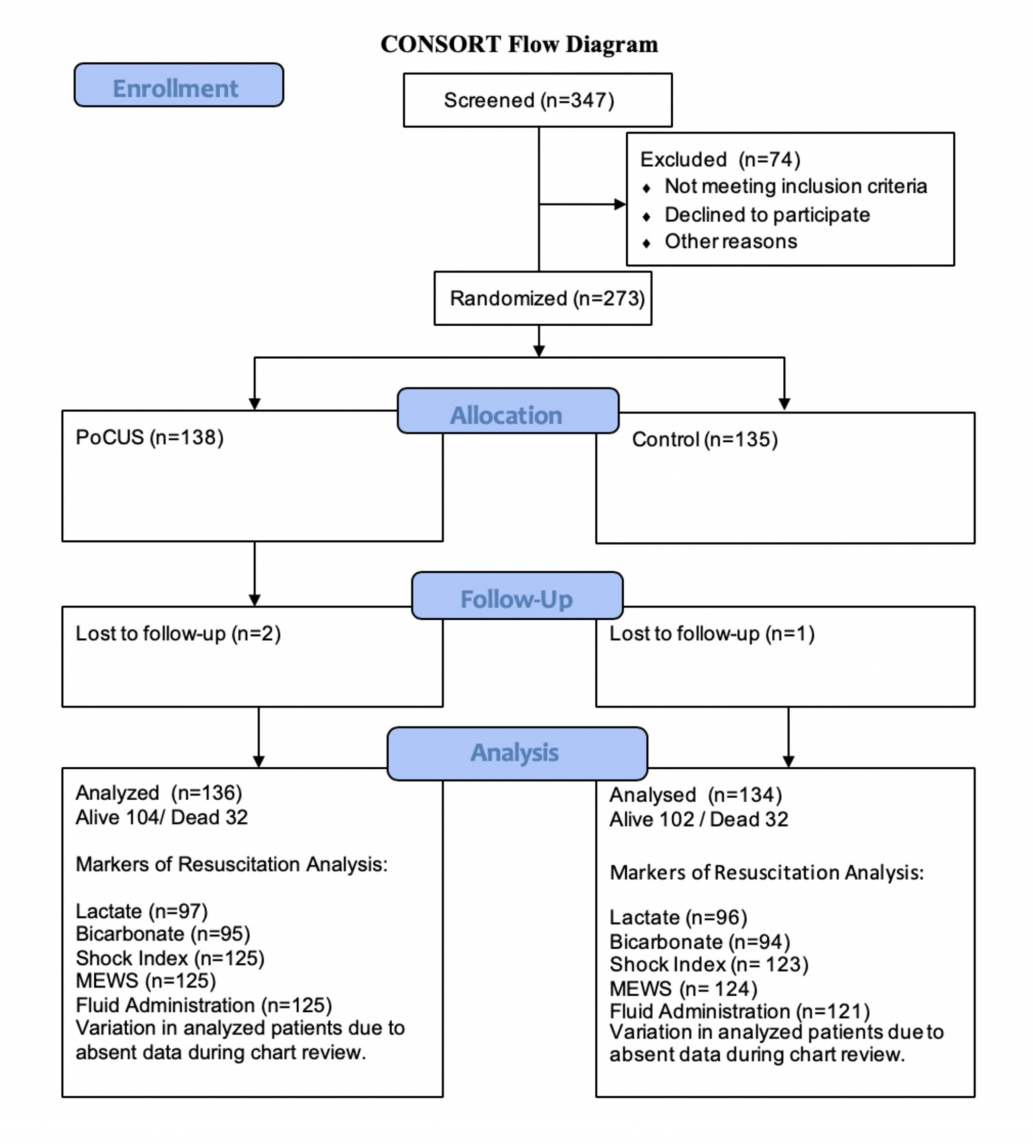
Consort Flow Diagram for SHoC-ED PoCUS: Point of Care Ultrasound; MEWS: Modified Early Warning Score

Patients were randomized to early PoCUS plus standard care, versus standard care without PoCUS (Figure 1).

**Figure 2:**
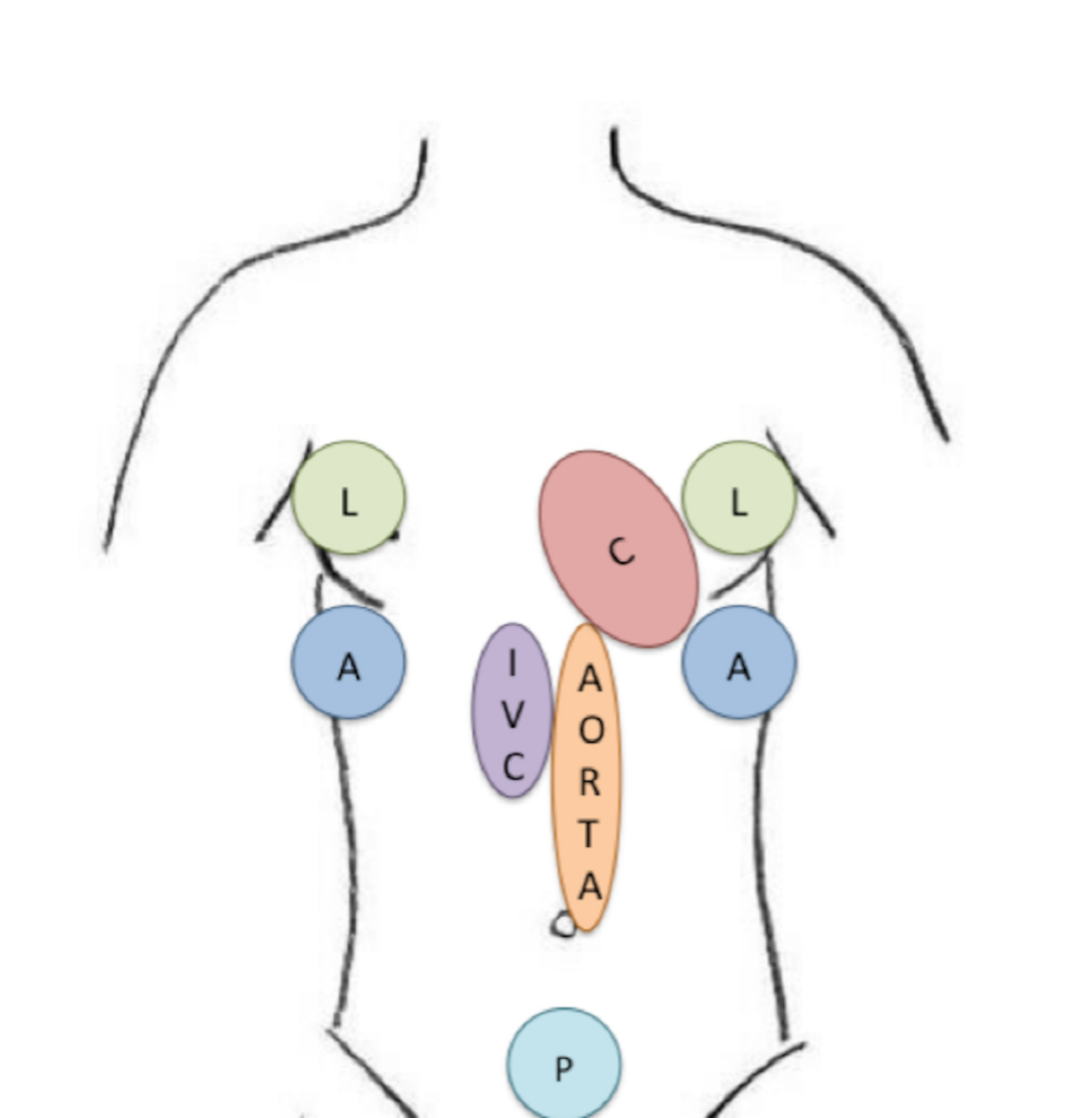
The sonographic protocol used consisted of a standardized shock-hypotension protocol based on a combination of the core components of the Abdominal and Cardio-thoracic Evaluation by Sonography for Shock (ACES) and Rapid Ultrasound in Shock and Hypotension (RUSH) protocols and was followed uniformly at all of the investigating sites. Cardiac (C) views included subxiphoid, parasternal long, parasternal short and apical views.  The presence or absence of pericardial fluid was noted as was left and right ventricular function and size.  Base of lung (thoracic) scans (L) were performed on the left and right side of the chest looking for the evidence of lung sliding to exclude tension pneumothoraces, and both pleural spaces were examined for pleural effusions.  The right and left upper quadrants of the abdomen (A) were examined for free fluid in the hepatorenal and spleno-renal regions. The inferior vena cava (IVC) was examined for size and collapsibility. The aorta (AORTA) was measured in a transverse and longitudinal plane to ascertain if an abdominal aneurysm was present. The pelvic views (P) were performed in the transverse and longitudinal planes to determine if free fluid was present in the peritoneal space, as well as an estimate of bladder filling. Adapted with permission from Atkinson et al. [1].

The PoCUS protocol (Figure 2), completed by trained physicians, consisted of a standardized shock-hypotension protocol, combining the core components of the Abdominal and Cardio-thoracic Evaluation by Sonography for Shock (ACES) and Rapid Ultrasound in Shock and Hypotension (RUSH) protocols [8,9]. Peripheral venous blood samples were drawn within the first hour in the ED and again at four hours. These were sent for venous blood gas analysis as well as serum lactate level. Vital signs required to determine the Shock Index (SI), and modified early warning score (MEWS) were prospectively recorded initially and again at four hours using standard data collection forms.

Previously reported outcomes included the primary outcome measure of survival to 30 days or hospital discharge, secondary outcome measures including the key interventions of initial intravenous (IV) fluid volume, frequency of inotrope administration, and frequency of recorded procedures, as well as investigations, admissions, and length of stay. Here we report shock index (SI), modified early warning score (MEWS), venous lactate and serum bicarbonate levels initially and at four hours expressed in mmol/L. Patients were analyzed based on their randomized groups: those who received PoCUS and those in the control group who did not receive PoCUS. The study was registered at ClinicalTrials.gov (registration number NCT01419106) and all sites received local research ethics board (REB) approval. A total study sample size of 265 provided a power of 0.80 (alpha 0.05) to detect a moderate effect size (>15%). Data were was analyzed using R software [R Core Team (2017), R Foundation for Statistical Computing, Vienna, Austria (https://www.R-project.org/)]. Study methods are outlined in further detail in the original report [1].

## Results

Baseline comparison

A total of 273 patients were enrolled across the six study sites. Data was collected for 270, with three patients being lost to follow up. Of those enrolled, 135 participants were randomized to the control group, and 138 to the PoCUS group (see the Consort flow diagram in Figure 1). Randomization was successful, with the groups being adequately matched for baseline demographics and vital signs, which also confirmed the shocked status of the patients with an overall mean systolic blood pressure of 91.2 (95% CI 89.5 to 93.0) mmHg, and a mean heart rate of 108.9 (95% CI 105.5 to 112.2) bpm (see Table 1). 

**Table 1:** Baseline demographic profile of study participants, and key outcomes. n: number; CI: confidence intervals; SBP: systolic blood pressure; HR: heart rate; MEWS: modified early warning score. Parts of this data have previously been reported by our group in Atkinson et al. [1].

Group characteristics and baseline measures		
Group	PoCUS	Control
Total Participants (n)	138	135
North America (n; %)	90 (65.2%)	89 (65.9%)
South Africa (n; %)	48 (34.8%)	46 (34.1%)
Male (n; %)	73 (52.9%)	65 (48.1%)
Age (years; Mean; 95% CI)	56.1 (53.0 to 59.3)	58.7 (55.5 to 61.9)
SBP (mmHg; Mean; 95% CI)	91.0 (88.7 to 93.4)	91.5 (88.9 to 94.2)
HR (bpm; Mean; 95% CI)	106.7 (102.0 to 111.3)	111 (106.0 to 116.0)
Resps (bpm; Mean; 95% CI)	24.2 (22.5 to 25.9)	23.7 (22.3 to 25.0)
Temp (deg C; Mean; 95% CI)	36.7 (36.5 to 36.8)	36.9 (36.6 to 37.1)
Outcomes		
Shock Index (mean reduction from baseline; 95%CI)	0.33 (0.29-0.38)	0.39 (0.34-0.44)
MEWS (mean reduction from baseline; 95%CI)	2.91 (2.49-3.32)	2.56 (2.22-2.89)
Lactate (mean reduction from baseline; 95%CI; mmol/L)	1.31 (0.88-1.74)	1.39 (0.93-1.85)
Bicarbonate (mean reduction from baseline; 95%CI; mmol/L)	2.30 (1.75-2.84)	2.71 (2.12-3.30)
Mean Fluid Bolus received (95%CI; ml)	1609 (1484-1732)	1658 (1510-1779)

Physiological scores

The Shock Index (SI) improved by a similar degree during initial treatment in the control and PoCUS groups (mean reduction in Control 0.39, 95% CI 0.34 to 0.44 vs. PoCUS 0.33, 0.29 to 0.38). The MEWS improved during resuscitation in each group, but with no meaningful difference between groups (mean reduction in Control 2.56, 2.22 to 2.89 vs. PoCUS 2.91, 2.49 to 3.32). 

Biochemical markers

There were similar mean reductions in bicarbonate (Control 2.71 mmol/L, 2.12 to 3.30 mmol/L vs. PoCUS 2.30 mmol/L, 1.75 to 2.84 mmol/L), and venous lactate (Control 1.39 mmol/L, 0.93 to 1.85 mmol/L vs. PoCUS 1.31 mmol/L, 0.88 to 1.74 mmol/L) levels in both groups over the course of the resuscitation. 

Fluid administration

Both groups received similar mean volumes of intravenous fluid during initial treatment (Control 1658 mL, 1510-1779 vs PoCUS 1609 mL, 1484-1732).

## Discussion

The secondary outcomes reported here, from this international multi-centre randomized controlled trial, are consistent with the previously reported findings that outcomes were similar for ED patients with undifferentiated shock independent of whether or not they received a PoCUS protocol. The improvements in both physiological scores and biochemical markers, along with similar volumes of intravenous fluid during initial treatment in both groups show that patients in each group received similar treatment and may help explain why no improvement in clinical outcomes such as survival or length of stay were seen [1]. 

As previously reported, the most common underlying cause of shock in both groups was sepsis. It is likely that clinicians resuscitated to similar clinical endpoints and targets, rather than to trends in PoCUS findings such as inferior vena cava (IVC) size or cardiac output measures [10]. 

These findings do not detract in any way from the widely accepted potential for PoCUS to detect critical diagnoses such as cardiac tamponade, aortic aneurysm or dissection, among others, but does support the notion that the singular use of a PoCUS protocol early in resuscitation, may not be sufficient to impact physiological improvements, in addition to clinical outcomes in this population. As discussed previously, the exclusion criteria used in this study likely blunted any potential impact of PoCUS by excluding patients who had a high clinical suspicion of critical diagnoses requiring immediate PoCUS for diagnoses. In addition, PoCUS was not routinely repeated to gauge the response to treatment. Also, the study was small and powered only to detect moderate differences between groups. We cannot exclude smaller differences. Finally, the heterogeneity in training may have resulted in instances where the physician was not able to generate conclusive views, negating some of the potential benefits for the intervention. This does however reflect real-world practice in that EDs are not fully staffed with ultrasound experts.

## Conclusions

In this randomized controlled trial, we did not find any clinically meaningful difference in physiological or biochemical resuscitation markers with or without the use of a point of care ultrasound protocol in the resuscitation of undifferentiated hypotensive emergency department patients. Both groups showed improvements in markers during the initial stages of resuscitation. These findings may help explain why no survival benefit was seen with PoCUS as previously reported. We caution against any conclusions relating to potential clinical benefits with PoCUS in individual patients or in specific shock types.
